# Optimal Plate Choice for High-Neck Mandibular Condyle Fracture: A Mechanistic Analysis of 16 Options

**DOI:** 10.3390/jcm13030905

**Published:** 2024-02-04

**Authors:** Jakub Okulski, Marcin Kozakiewicz, Michał Krasowski, Rafał Zieliński, Piotr Szymor

**Affiliations:** 1 Department of Maxillofacial Surgery, Medical University of Lodz, 113 Żeromskiego Str., 90-549 Lodz, Poland; jakub.okulski@gmail.com (J.O.); marcin.kozakiewicz@umed.lodz.pl (M.K.); bkost@op.pl (R.Z.); 2 Material Science Laboratory, Medical University of Lodz, 251 Pomorska Str., 92-213 Lodz, Poland; michal.krasowski@umed.lodz.pl

**Keywords:** high-neck surgical treatment, ORIF, condylar fractures, MEF

## Abstract

(1) **Background**: Mandibular fractures are common, with the condylar process being a frequent site of injury, accounting for 25–45% of cases. This research aims to assess the mechanical suitability of various plates for high-neck condyle fractures. (2) **Methods**: Polyurethane models mimicking high-neck condyle fractures were utilized in this study. Sixteen distinct plate designs, constructed from titanium sheets, were tested. The figures underwent force assessments on a durability testing apparatus, and the relationship between used force and fracture movement was documented. (3) **Results**: For high-neck breaking, the two straight plates emerged as the most effective, aligning with established osteosynthesis standards. The second-best plate exhibited nearly half the strength of the gold standard. (4) **Conclusions**: In response to the aim of this study, considering the mechanical aspects, the double plain plate stands out as the optimal choice for osteosynthesis in cases of high-neck fractures of the mandibular condylar process. In addition, the authors propose the Mechanical Excellence Factor (MEF) as a superior metric for appraising a plate’s mechanical force, surpassing the conventional Plate Design Factor (PDF).

## 1. Introduction

Of the entire craniofacial skeleton, the mandible is the most vulnerable to injury. However, as many as 8% of fractures involve the high condylar necks of the mandible [1,2,3].

There are two methods of treating mandibular fractures: conservative treatment and surgical treatment. Upon reviewing the literature, there is no clear consensus on the optimal treatment method [4,5,6,7,8]. Each method has its own advantages and disadvantages, with indications for treatment depending on factors such as age, comorbidities, and the patient’s general condition. The authors acknowledge the advantages of surgical treatment, particularly in condylar process fractures associated with mandibular ramus height shortening and/or fragment rotation.

The advantages of surgical treatment include the ability to achieve proper and permanent fracture stabilization, especially in multifracture mandibular cases, a quicker return to normal functioning in society with no speech or eating impediments (only requiring a liquid diet), and no restrictions on upper airway access in polytrauma patients. Studies [5] demonstrate that patients treated surgically experience greater mandibular retraction and fewer occurrences of abnormal occlusion compared to conservative treatment.

Surgical treatment presents clinical challenges due to limited access and proximity to important anatomical structures, such as the maxillary artery, mandibular vein, facial nerve, trigeminal nerve, and articular surface [9]. Additionally, post-operative complications often arise from fixation instability, including plate fractures or screw loosening.

Conservative treatment is typically inadequate unless the bone fragments are axially aligned (rotation less than 30 degrees) and there is no significant mandibular ramus height shortening (less than 2 mm) [9]. However, this represents a minority of patient cases based on the authors’ clinical experience.

In the ideal scenario of osteosynthesis, two primary lines are deemed best for positioning a plate. The initial line follows parallel to the arch between the condylar process and the coronoid process. Osteosynthesis at this location counteracts the tensile forces during mastication. The second line runs parallel to the line between the condylar process and the angle of the mandible. Osteosynthesis at this location counteracts the resulting bending stresses in the frontal plane and rotational stresses in the axial plane [10].

Using two straight plates has been established as the optimal method for managing high-neck condylar fractures of the mandible. It could be reasonable to emphasize that smaller plates may present a viable alternative to using two straight plates, considering the limited visibility and challenging surgical access to the neck of the mandibular condyle.

This study seeks to assess and compare the mechanical effectiveness of numerous plates employed in managing mandibular high-neck breakage.

## 2. Materials and Methods

Various types of plates are utilized for bone osteosynthesis following craniofacial trauma. For upper and middle facial fractures (e.g., craniofacial lid bones, orbit, zygomatic and maxillary bones), plates from the 1.2, 1.5, and 1.8 systems are commonly employed. For the lower facial floor, specifically for mandibular trauma fractures, 2.0 system plates are used. In oncological procedures involving segmental mandible resection, 2.7 system plates are utilized.

The study obtained 51 plate designs for mandibular fracture osteosynthesis in the 2.0 system from various sources, including plate manufacturers (ChM, KLS Martin, Global D, Synthes DePuy, and Medartis) and designs from the Medical University of Lodz, Poland. Sixteen plate designs were selected for the osteosynthesis of high-neck fractures including two criteria sizes (system 2.0) and the available osteosynthesis space. These designs were created using CAD software (SolidWorks 2019 SP2) and laser cut from medical-grade titanium plates (grade 23, 1 mm thick) to enhance ductility and fracture toughness compared to titanium grade 5 [11]. All of the plate designs we compared were cut from a flat sheet of titanium and were not bent to the anatomical shape of the fracture. Prior to the study, statistical tests had not previously been performed to determine the number of tests needed per plate to detect statistically significant differences in the performance of the plates analyzed.

We used polyurethane standardized mandibular models that follow the guidelines set forth in the literature and the recommendations of the American Society for Testing and Materials (ASTM) for testing orthopedic implants biomechanically [12]. These models are made of a material that mimics the properties of spongy bone in humans. Specifically, for strength assessments, we employed polyurethane mandibles (Sawbones, Vashon, WA, USA: density 0.16 g/cc, compression modulus 58 MPa) [13,14,15,16,17]. 

Each mandibular condylar process underwent simulation of a high-neck fracture by executing an incision, conforming to Kozakiewicz’s categorization of mandibular condylar process fractures [9]. However, our study predominantly adhered to the AO Foundation classification, which enjoys broader recognition [18].

**Figure 1:**
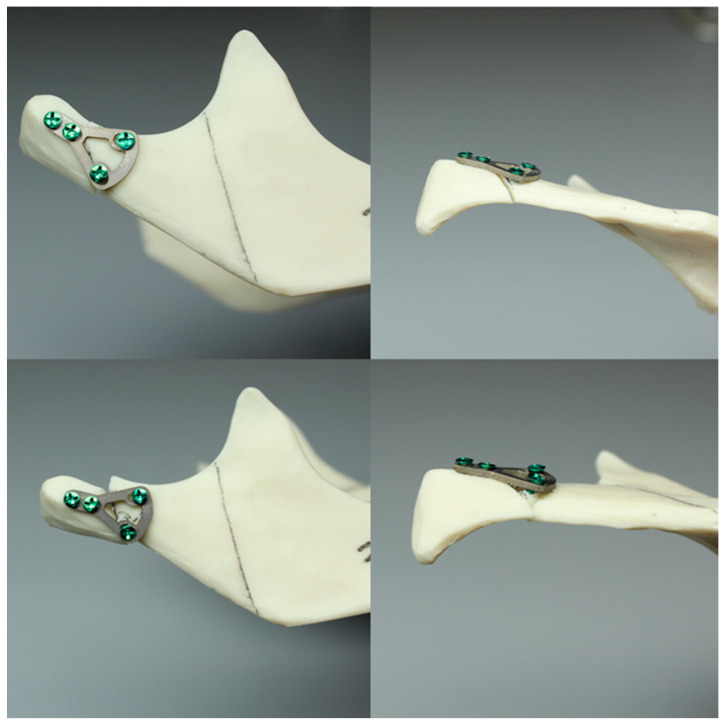
Mandible model before (two top photos) and after testing (two bottom photos).

Next, the two segments—the condylar (proximal) and the ramus (distal)—were joined together to simulate the realignment of fragments as carried out in surgical procedures. A plate was positioned over these fragments, and the mandibular model was drilled at the precise locations matching the holes in the plate, using a calibrated 1.5 mm diameter drill. The drilling process involved guiding the drill perpendicular to the surface of the bone. Subsequently, the plate was secured using self-tapping of certified titanium screws, which were 6.0 mm in length and 2.0 mm in diameter. During screwing, the plates were tightened so that they kept their shape, that is, so that they did not bend. Each plate type underwent the fixation process for the fracture seven times (as shown in Figure 1).

In order to simulate pressure in the condylar region during food consumption, the condyle was positioned at an angle of 10° in the frontal plane and 15° in the sagittal plane. To achieve this, the mandible was rigidly fixed on a special base in the shape of a truncated cuboid. Forces in upward, forward, and medial directions were applied to the condylar by a machine piston. This configuration was aimed at closely imitating the muscle forces exerted on the condylar process during the act of chewing.

**Figure 2:**
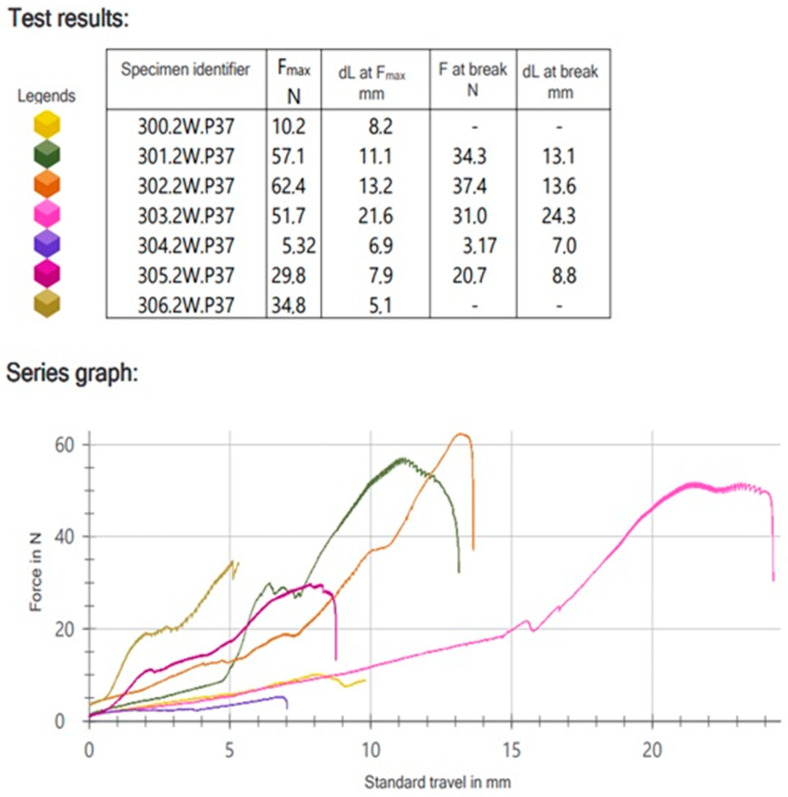
Graph from testXpert II V3.31.

The entirety of the assessments was conducted by the authors at the Material Science Laboratory situated at the Medical University of Lodz in Poland. For the force evaluations, a Zwick Roell Z020 common testing apparatus, manufactured by Zwick-Roell based in Ulm, Germany, was utilized. Initially, a force of 1 N was applied to the condyle, gradually increasing at a rate of 1 mm per minute in correlation with the downward movement of the piston. Testing ceased upon occurrence of a plate fracture, condyle breakage, screw detachment, or when the system experienced significant deformation causing the condyle to contact the machine base. The results were recorded using dedicated Instron software, testXpert II V3.31, specifically designed for the Zwick Roell machine. The following parameters were logged, including the relationship betwixt applied strength (Fmax [N]) and piston movement (dL at Fmax [mm]) (as depicted in Figure 2).

The PDF (Plate Desing Factor) [19] and MEF (Mechanical Excellence Factor) [17] already recommended by the authors were determined for each plate (Table 1).

The statistical investigation was carried out using Statgraphics Centurion 18 software developed by Statgraphics Technologies Inc., located in Warrenton, VA, USA. Between-design comparisons were conducted employing either ANOVA or the Kruskal–Wallis test. Categorical variables were assessed using independent χ2 tests. Linear regression analysis was employed to evaluate the relationship between the two quantitative variables. Determining the optimal plate design was based on an objective description. Analytical implication was specified as a *p*-value below 0.05.

## 3. Results

Table 1 shows the test outcomes for all of the 16 plate patterns. The average strength needed to move the condylar process attachment by 1 mm was recorded as 6.12 ± 2.98 N, with a median value of 5.69 N. Across the study group, the maximum force needed to reposition the condyle by 1 mm was 14.02 ± 1.24 N. This outcome significantly differs from the force observed in the unbroken mandibular model, which was calculated to be 28.33 ± 3.16 N (Mann–Whitney (Wilcoxon) test; *p* < 0.001).

**Figure 3:**
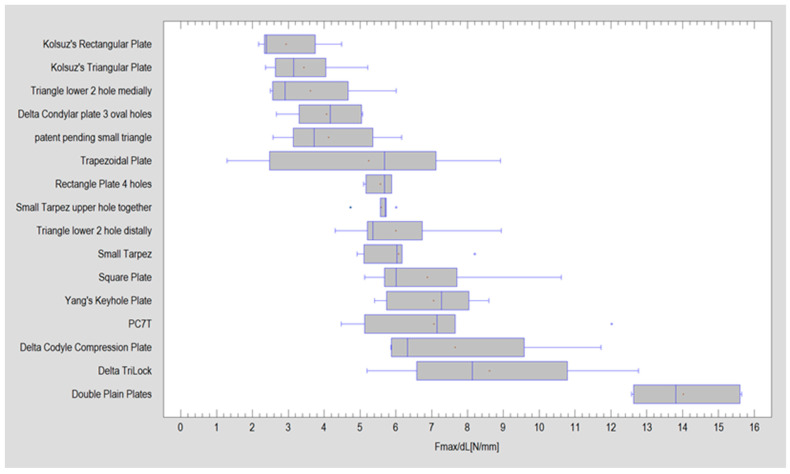
Results from the experiment are illustrated for all plate patterns, showcasing the strength required for a one-millimeter movement of the immovable parts. The mean is represented by a red cross, and the median is depicted by a thin vertical line inside the grey box. Blue points on the graph indicate Fmax/dL for a specific plate, with values outside the standard deviation.

The outcomes regarding the force necessary to displace the fixation by 1 mm exhibited noteworthy differences among whole analyzed plate patterns (Kruskall–Wallis test; *p* < 0.001). The most substantial result was observed in the case of two-plate osteosynthesis utilizing straight plates (14.02 ± 1.24 N). Notably, the force required to displace the fixation using the second strongest plate was almost half that of the top-performing plate (Figure 3 and Table 2).

**Figure 4:**
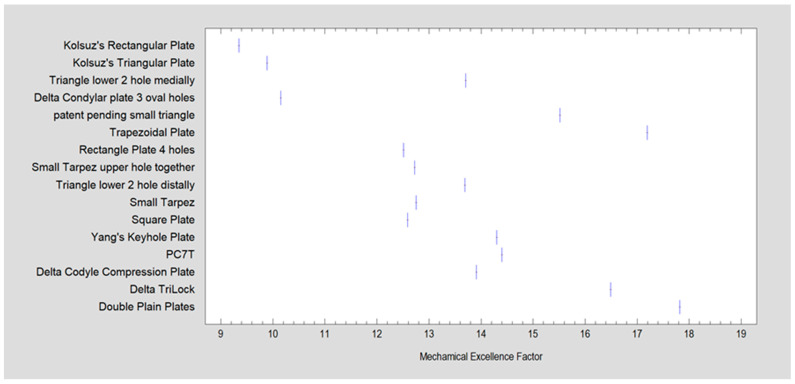
Relation betwixt plate and Mechanical Excellence Factor (MEF).

Figure 4 depicts the correlation betwixt the plate and Mechanical Excellence Factor (MEF), represented by tight vertical lines in the picture. The overall tendency between the MEF and plate design indicates an unsatisfactory fit.

**Figure 5:**
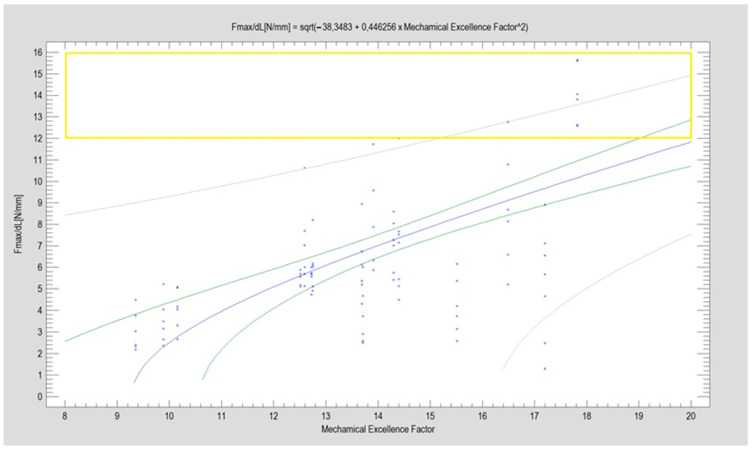
The correlation between the cumulative assessment of plate construction, measured by the Mechanical Excellence Factor (MEF), and the resilience to traverse force of stabilized parts in high-neck breakage of the mandibular condylar process is statistically significant (*p* < 0.001). The relationship between these two characteristics is moderately strong, with a correlation coefficient (CC) of 0.59. The mathematical description model presented in this study accounts for a minority of observations (*R*^2^ = 35%). The regression equation plot is depicted by the blue line, where each point denotes an experimental result obtained. The precise load test results are detailed in Table 1. Confidence limits, indicated by green lines, are established at a 95% confidence level, while prediction limits are represented by gray lines. Notably, a group of designs showcasing promising construction features (MEF > 20) is not readily identifiable. However, a discernible group of designs capable of providing high stability in fixation (Fmax/dL > 12 N) is highlighted within the yellow box.

Plate patterns exhibiting an MEF higher than 20 are categorized as encouraging plate constructions. However, such high MEF-rated plates were not found for this particular type of fracture. In this study, only small-sized plates were examined, which contrasts with the plates assessed in condylar basal or low-neck fractures. Designs that underwent testing and demonstrated a strength in moving elements of 1 mm above 12 N were categorized as robust plate patterns. The data encompass all experimental results gathered from 112 experiments (Figure 5).

## 4. Discussion

Biomechanic test outcomes can be affected by differences in bone density and elastic modulus [21,22]. An optimal test model should closely mimic human bone characteristics. However, achieving clarity in test outcomes is challenging due to factors such as the thickness and quality of both the compact lamina and spongy part of the bone. These aspects are influenced by bone mineralization levels, which, in turn, depend on various factors like dietary intake, including vitamin D3 and calcium supplementation, hormone levels, and medications. Medications (for example, bisphosphonates) used for conditions like osteoporosis, prostate cancer, breast cancer, and bone metastases can significantly impact bone quality and density. Consequently, conducting tests using human cadaver bones also presents limitations and is far from an ideal solution.

Another avenue for research lies in animal studies [23,24,25,26,27,28], which is a well-established research methodology. However, there are inherent limitations in this approach. Variations in the mandible’s shape across different animal species result in distinct stress distributions, impacting the relevance of findings. Moreover, dietary differences among animals like sheep, cats, mini pigs, or rodents also contribute to deviations from ideal conditions for the study. Additionally, conducting studies involving a larger animal cohort poses logistical challenges, making such investigations more complex and demanding.

Another approach involves the Finite Element Method (FEM) testing of the plates, a commonly utilized method in preclinical studies [29]. However, there are certain drawbacks associated with this method. FEM testing has the potential to create scenarios that are overly idealistic, potentially leading to results that may not accurately reflect clinical realities. To execute such studies effectively, the research team requires substantial expertise, along with sufficient funding and time to develop a model that comprehensively encapsulates all relevant variables.

Another potential option involves generating mandible models through 3D printing, enabling the replication of both the compact lamina and spongy bone. However, this approach was dismissed by the authors due to certain challenges. Standardizing these models, ensuring they strike a balance between being neither too fragile nor too rigid compared to human mandibles, posed difficulties [30]. Additionally, the lengthy production time and high costs associated with this method led to its rejection by the authors.

The study opted for polyurethane, a frequently used material in orthopedic research. However, it is important to note that the polyurethane model does not replicate the cortical structure of bone, which plays a crucial role in the primary stabilization of screws during osteosynthesis. Instead, this model comprises a uniformly dense filler resembling spongy bone. This difference in structure might potentially impact the test results obtained.

As highlighted in Table 1, the double plain plate (Plate 20, considered the gold standard) emerges as the most effective option. Notably, despite variations in plate shapes, the top performer remains the configuration with two straight plates. The plate ranking second is nearly half as robust as the acknowledged gold standard. This discrepancy could potentially be attributed to the plates’ ability to be placed at a distance from each other, enabling alignment along stress lines and consequently reducing the impact of torsional forces. Plate 20, in comparison to plate 46, lacks oval holes which, according to MEF calculations, can diminish fixation efficiency. While none of the plates achieved the targeted MEF > 20, Plate 20 notably attained a commendable level of fixation stability (Fmax/dL > 12 N) compared to others, as evident in Figure 5 and Table 1. Potential other aspects of the mechanical construction of the plates that could lead to low results include whether there is a gap between the holes (Plate 20) or not (Plate 46), whether the oval hole occurs independently on one arm (Plate 48) or together with a circular hole (Plate 47), whether the holes are positioned one after the other (Plate 34) or if there is a gap between them (Plate 31), and the distance of hole placement from the fracture line.

In a previous publication, the authors proposed the MEF factor as a means to expedite the validation process of new osteosynthesis plate designs. This factor integrates eight structural features and represents an enhancement over the previously introduced PDF (Plate Design Factor), which assessed only four features. The MEF has shown efficacy in anticipating the success of plate fixation in basal fractures and low-neck fractures.

However, as illustrated in Figure 4 and Figure 5 regarding high-neck fractures, the MEF factor does not demonstrate effectiveness in predicting the success of fixation using a specific plate in these scenarios. This discrepancy suggests limitations in the MEF factor’s applicability for high-neck fractures, contrasting with its reliability in other types of fractures. Potential reasons that the MEF factor does not perfectly predict plate behavior may be because it does not take into account the following elements: number of screws in the ramus, number of oval holes in the ramus, number of oval holes in the condyle, and plate thickness between holes (Plate 51 vs. Plate 34)

Some researchers [31] argue that the frequency of reoperations is not influenced by the type of plate utilized, with most reoperations attributed to factors such as improper alignment of bone fragments, inadequate fusion of bone segments, subsequent mandibular dislocation, and hematoma in the operated region. Furthermore, according to their findings, the occurrence of loosened osteosynthesis screws is not contingent on the plate shape but rather on factors such as compromised bone quality, bilateral condylar fractures, challenges in precise plate placement due to limited surgical access, the trajectory of the fracture line, and the presence of intermediate fragments.

Conversely, our standardized studies (ensuring consistent bone density, identical fracture lines, ease of fragment positioning, and no issues with plate placement) demonstrate significant variations in plate strength based on plate shape. Moreover, our research suggests a correlation between the number of screws in the plate and the stability of fixation, indicating that a higher number of screws enhances stability. These findings contrast with the conclusions drawn by the aforementioned authors regarding the influence of plate shape and screw fixation on stability.

Resorbable plates offer the distinct advantage of self-dissolving properties, eliminating the necessity for a subsequent surgery for plate removal in pediatric cases. Additionally, in adult patients, the use of resorbable plates mitigates the risk of potential inflammation in the long term. Some studies suggest that resorbable plates exhibit a comparable strength to titanium plates [32], implying their potential suitability as a viable alternative. However, other research emphasizes the necessity for clinical trials to substantiate and confirm their effectiveness [33]. These differing perspectives underscore the need for further investigation and empirical evidence to ascertain the reliability and efficacy of resorbable plates in clinical settings.

The test results presented by the authors suggest which plate could potentially offer the most clinical effectiveness. However, it is crucial to acknowledge that these findings stem solely from strength tests and lack consideration for biological variables. For a comprehensive assessment of a particular plate’s efficacy, future evaluations should entail selecting plates post-mechanical testing and subjecting them to strength–fatigue tests. Following this, the identified plates should proceed to undergo clinical trials. Clinical trials hold substantial significance in determining the optimal plate, especially considering the current high reoperation rates (up to 16%) attributed to plate fractures [31]. Nevertheless, it is important to note that new plate models continually emerge, some of which have not been investigated by the authors [34]. It should also be noted that the treatment of high-neck fractures is still a challenge for operators. The literature also describes cases of making individual implants for osteosynthesis of this type of fracture [35]. This underscores the need for ongoing research and evaluations to keep pace with advancements and continuously improve treatment outcomes in this field.

The article complements existing literature as the number of studies comparing plates for the osteosynthesis of high-neck fractures is limited. One of them relates to research conducted earlier by the authors, in which four plates for high-neck fractures were compared. In this study, the research was expanded by an additional 12 plate designs and the MEF coefficient.

Certainly, this study has several limitations that should be acknowledged. Firstly, the utilization of polyurethane models and specific plate designs might restrict the generalizability of our findings. The properties of polyurethane models may not precisely mirror those of human bone, and the outcomes might differ when considering plates crafted from various materials or featuring diverse patterns. Additionally, the analysis focuses on a particular group of condylar process breakages, as per the Kozakiewicz classification, which could probably impact the outcomes. Variations in fracture types and classifications might yield different outcomes, limiting the broader applicability of our findings beyond this specific fracture classification. Therefore, while our study provides valuable insights into plate performance under specific conditions, these limitations underscore the necessity for further research encompassing a broader range of bone models, plate designs, and fracture classifications to enhance the comprehensiveness and applicability of our conclusions to diverse clinical scenarios.

## 5. Conclusions

In addressing the question posed in the introduction of this publication, from a mechanical standpoint, the double plain plate emerges as the most suitable option for the osteosynthesis of high-neck fractures of the mandibular condylar process. Utilizing this plate significantly minimizes the risk of fixation loss, showcasing superior stability compared to other options. However, it is important to note that while the Mechanical Excellence Factor (MEF) factor is effective in predicting the success of fixation in basal and low-neck fractures, our findings suggest its limited effectiveness in predicting fixation success specifically in high-neck fractures. Therefore, relying solely on MEF for prognosis in high-neck fractures may not be accurate or reliable.

## Figures and Tables

**Table 1:** The plated designs are arranged in ascending order based on the strength of the fixing material, ranging from most powerless to most powerful.

Name	Design Code	Design	H [mm]	W [mm]	S [mm^2^]	PDF	MEF	Fmax/dL [N/mm]
Kolsuz’s rectangular plate	Plate 48	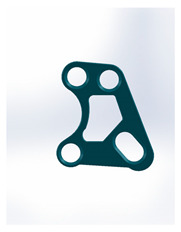	11.4	10.1	49	67	9.4	2.94 ± 0.88
Kolsuz’s triangular plate	Plate 47	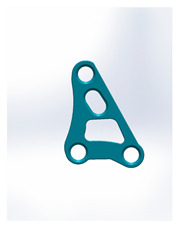	14	10.4	58	79	9.9	3.43 ± 0.96
Triangle lower 2 holes medially	Plate 45	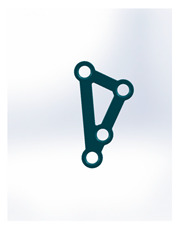	18.1	9.8	67	90	13.7	3.61 ± 1.31
Delta condylar plate 3 oval holes	Plate 37	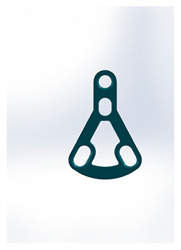	17	11.4	64	87	10.2	4.07 ± 0.87
Patent pending small triangle *	Plate 11	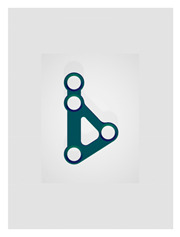	13.5	8	138	151	15.5	4.12 ± 1.25
Trapezoidal plate	Plate 51	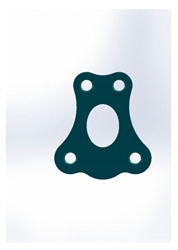	20	18	189	213	17.2	5.24 ± 2.66
Rectangular plate 4 holes	Plate 32	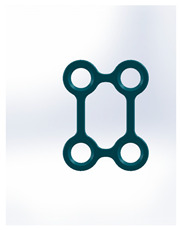	11	8.5	37	55	12.5	5.57 ± 0.32
Small trapeze upper hole together	Plate 34	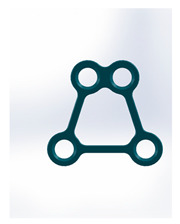	11.1	12.1	40	61	12.7	5.60 ± 0.40
Triangle lower 2 holes distally	Plate 42	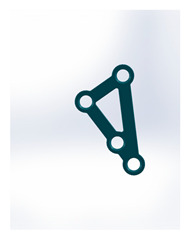	17.8	10	67	90	13.7	6.00 ± 1.50
Small trapeze	Plate 33	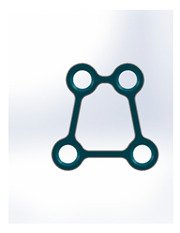	11.6	11.6	41	61	12.8	6.08 ± 1.06
Square plate	Plate 31	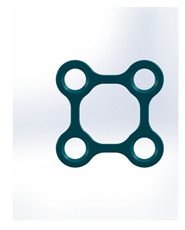	10	10	39	57	12.6	6.88 ± 1.85
Yang’s keyhole plate	Plate 46	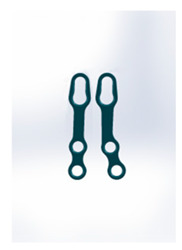	19.7	9.9	80	106	14.3	7.05 ± 1.15
PC7T	Plate 49	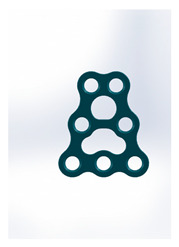	15	12.8	86	110	14.4	7.06 ± 2.52
Delta condyle compression plate *	Plate 14	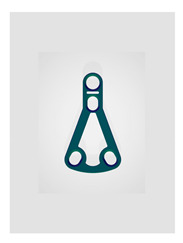	15.3	8.8	179	191	13.9	7.65 ± 2.24
Delta TriLock *	Plate 02	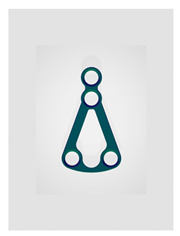	15.4	8.8	174	187	16.5	8.61 ± 2.52
Double plain plates *	Plate 20	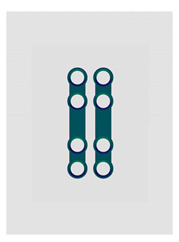	16.5	3.4	227	236	17.8	14.02 ± 1.24

* Plate patterns correlate to the shapes given in a previous publication [20]. Abbreviations: H—height [mm], W—width [mm], S—surface area [mm^2^], PDF—Plate Design Factor, MEF—Mechanical Excellence Factor.

**Table 2:** Various coverage tests were conducted to assess the force required for moving the attachment by 1 mm (Fmax/dL, N) based on the plate used.

Homogenous Groups ^1^	Mean Fmax/dL	Name
X	2.94	Kolsuz’s rectangular plate
X	3.43	Kolsuz’s triangular plate
XX	3.61	Triangle lower 2 holes medially
XXX	4.07	Delta condylar plate 3 oval holes
XXX	4.12	Patent pending small triangle
XXX	5.24	Trapezoidal plate
XXX	5.57	Rectangle plate 4 holes
XXX	5.6	Small trapeze upper hole together
XXX	6	Triangle lower 2 holes distally
XXX	6.08	Small trapeze
XXX	6.88	Square plate
XXX	7.05	Yang’s keyhole plate
XXX	7.06	PC7T
XX	7.65	Delta condyle compression plate
X	8.61	Delta TriLock
X	14.02	Double plain plates

^1^ In the given column, the stages marked with X’s constitute a group of means where there are no numerical significant aberrations. The procedure employed to discriminate among the means is Fisher’s least significant difference (LSD) procedure. Using this procedure, there is a 5.0% risk of incorrectly determining each pair of means as significantly different when the actual variation is zero.

## Data Availability

The data presented in this study are available on request from the corresponding author. The data are not publicly available due to an ongoing multicentre project.

## References

[1] Al-Moraissi E.A., Ellis E. (2015). Surgical treatment of adult mandibular condylar fractures provides better outcomes than closed treatment: A systematic review and meta-analysis. J. Oral Maxillofac. Surg..

[2] Chrcanovic B.R. (2015). Surgical versus non-surgical treatment of mandibular condylar fractures: A meta-analysis. J. Oral Maxillofac. Surg..

[3] Kozakiewicz M., Walczyk A. (2023). Current Frequency of Mandibular Condylar Process Fractures. J. Clin. Med..

[4] Durmuş Kocaaslan N., Karadede Ünal B., Çavuş Özkan M., Karadede B., Çelebiler Ö. (2022). Comparison of different treatment techniques in the mandibular condyle fracture. Turk. J. Trauma Emerg. Surg..

[5] Yao S., Zhou J., Li Z. (2014). Contrast analysis of open reduction and internal fixation and non-surgical treatment of condylar fracture: A meta-analysis. J. Craniofac. Surg..

[6] Shiju M., Rastogi S., Gupta P., Kukreja S., Thomas R., Bhugra A.K., Parvatha Reddy M., Choudhury R. (2015). Fractures of the mandibular condyle—Open versus closed—A treatment dilemma. J. Craniomaxillofac. Surg..

[7] Al-Moraissi E.A., Neff A., Kaur A., Falci S.G.M., de Souza G.M., Ellis E. (2023). Treatment for Adult Mandibular Condylar Process Fractures: A Network Meta-Analysis of Randomized Clinical Trials. J. Oral Maxillofac. Surg..

[8] Karan A., Kedarnath N.S., Reddy G.S., Harish Kumar T.V.S., Neelima C., Bhavani M., Nayyar A.S. (2019). Condylar Fractures: Surgical Versus Conservative Management. Ann. Maxillofac. Surg..

[9] Kozakiewicz M. (2019). Złamania Wyrostka Kłykciowego Żuchwy.

[10] Shakya S., Zhang X., Liu L. (2020). Key points in surgical management of mandibular condylar fractures. Chin. J. Traumatol..

[11] https://www.kronosedm.pl/tytan-r56401/.

[12] (2016). Standard Specification for Rigid Polyurethane Foam for Use as a Standard Material for Testing Orthopaedic Devices and Instruments.

[13] Assari S., Darvish K., Ilyas A.M. (2012). Biomechanical analysis of second-generation headless compression screws. Injury.

[14] Baran O., Sagol E., Oflaz H., Sarikanat M., Havitcioglu H. (2009). A biomechanical study on preloaded compression effect on headless screws. Arch. Orthop. Trauma. Surg..

[15] Ramaswamy R., Evans S., Kosashvili Y. (2010). Holding power of variable pitch screws in osteoporotic, osteopenic and normal bone: Are all screws created equal?. Injury.

[16] Bailey C.A., Kuiper J.H., Kelly C.P. (2006). Biomechanical Evaluation of a New Composite Bioresorbable Screw. J. Hand Surg..

[17] Kozakiewicz M., Okulski J., Krasowski M., Konieczny B., Zieliński R. (2023). Which of 51 Plate Designs Can Most Stably Fixate the Fragments in a Fracture of the Mandibular Condyle Base?. J. Clin. Med..

[18] Mittermiller P.A., Bidwell S.S., Thieringer F.M., Cornelius C.P., Trickey A.W., Kontio R., Girod S., AO Trauma Classification Study Group (2019). The Comprehensive AO CMF Classification System for Mandibular Fractures: A Multicenter Validation Study. Craniomaxillofac. Trauma Reconstr..

[19] Kozakiewicz M., Zieliński R., Krasowski M., Okulski J. (2019). Forces Causing One-Millimeter Displacement of Bone Fragments of Condylar Base Fractures of the Mandible after Fixation by All Available Plate Designs. Materials.

[20] Zieliński R., Kozakiewicz M., Konieczny B., Krasowski M., Okulski J. (2020). Mechanical Evaluation of Titanium Plates for Osteoesynthesis High Neck Condylar Fracture of Mandible. Materials.

[21] Goldstein S. (1987). The mechanical properties of trabecular bone: Dependence on anatomic location and function. J. Biomech..

[22] Chapman J.R., Harrington R.M., Lee K.M., Anderson P.A., Tencer A.F., Kowalski D. (1996). Factors Affecting the Pullout Strength of Cancellous Bone Screws. J. Biomech. Eng..

[23] Schindeler A., Mills R.J., Bobyn J.D., Little D.G. (2018). Preclinical models for orthopedic research and bone tissue engineering. J. Orthop. Res..

[24] Cutcliffe H.C., DeFrate L.E. (2021). Four-Point Bending Testing for Mechanical Assessment of Mouse Bone Structural Properties. Methods Mol. Biol..

[25] Alkan A., Metin M., Muğlali M., Ozden B., Celebi N. (2007). Biomechanical comparison of plating techniques for fractures of the mandibular condyle. Br. J. Oral Maxillofac. Surg..

[26] Pilling E., Eckelt U., Loukota R., Schneider K., Stadlinger B. (2010). Comparative evaluation of ten different condylar base fracture osteosynthesis techniques. Br. J. Oral Maxillofac. Surg..

[27] Kot C.C.S., Verstraete F.J.M., Garcia T.C., Stover S.M., Arzi B. (2022). Biomechanical evaluation of locking versus nonlocking 2.0-mm malleable L-miniplate fixation of simulated caudal mandibular fractures in cats. Am. J. Vet Res..

[28] Olivera L.B., Sant’ Ana E., Manzato A.J., Guerra F.L., Arnett G.W. (2012). Biomechanical in vitro evaluation of three stable internal fixation techniques used in sagittal osteotomy of the mandibular ramus: A study in sheep mandibles. J. Appl. Oral Sci..

[29] Cheng C.K., Wang X.H., Luan Y.C., Zhang N.Z., Liu B.L., Ma X.Y., Nie M.D. (2019). Challenges of pre-clinical testing in orthopedic implant development. Med. Eng. Phys..

[30] Marturello D.M., Wei F., Déjardin L.M. (2019). Characterization of the torsional structural properties of feline femurs and surrogate bone models for mechanical testing of orthopedic implants. Vet Surg..

[31] Sikora M., Chęciński M., Nowak Z., Chęcińska K., Olszowski T., Chlubek D. (2021). The Use of Titanium 3D Mini-Plates in the Surgical Treatment of Fractures of the Mandibular Condyle: A Systematic Review and Meta-Analysis of Clinical Trials. J. Clin. Med..

[32] Agnihotry A., Fedorowicz Z., Nasser M., Gill K.S. (2017). Resorbable versus titanium plates for orthognathic surgery. Cochrane Database Syst. Rev..

[33] Dorri M., Oliver R. (2018). WITHDRAWN: Resorbable versus titanium plates for facial fractures. Cochrane Database Syst. Rev..

[34] Fuessinger M.A., Gass M., Woelm C., Cornelius C.P., Zimmerer R.M., Poxleitner P., Schlager S., Metzger M.C. (2021). Analyzing the Fitting of Novel Preformed Osteosynthesis Plates for the Reduction and Fixation of Mandibular Fractures. J. Clin. Med..

[35] Kawai T., Kawamata S., Suzuki S., Ishikawa Y., Ikeda Y., Yasuge E., Kogi S., Ogawa A., Izumisawa M., Yamada H. (2023). Condyle Fracture Fixed with Custom-Made Titanium Mesh and a Miniplate: A Case Report. Am. J. Case Rep..

